# Fatty Acids and Free Amino Acids Changes during Processing of a Mediterranean Native Pig Breed Dry-Cured Ham

**DOI:** 10.3390/foods9091170

**Published:** 2020-08-25

**Authors:** Eva Salazar, José Mª Cayuela, Adela Abellán, Estefanía Bueno-Gavilá, Luis Tejada

**Affiliations:** Departamento de Tecnología de la Alimentación y Nutrición, Universidad Católica de Murcia, Campus de los Jerónimos, Guadalupe, 30107 Murcia, Spain; jmcayuela@ucam.edu (J.M.C.); aabellan@ucam.edu (A.A.); ebueno@ucam.edu (E.B.-G.); ltejada@ucam.edu (L.T.)

**Keywords:** quality for food processing, pig, native species, fatty acids, free amino acids, dry-cured ham

## Abstract

The aim of this work is to analyse the intramuscular fatty acids and the free amino (FAA) acids in Chato murciano dry-cured ham. There are several Mediterranean native pig breeds whose characteristics of derived products have been described, but the impact of lipolysis and proteolysis on Chato murciano dry-cured ham has not yet been studied. Fatty acids and free amino acids were determined in the fresh piece and at 14, 18, 22 and 24 months of manufacturing. Monounsaturated fatty acids are the majority in the neutral lipids and free fatty acid fractions. Lipolysis took place mainly until the 18th month, resulting in a decrease in the levels of fatty acids of neutral lipids (from 95.43% to 83.38%) and polar lipids (from 2.57% to 0.41%), accompanied by a corresponding increase in free fatty acids (from 2% to 16.21%). Neutral lipids hydrolysis provides the main free fatty acids as in other native breeds. Results for FAA showed an increase in concentration during the time preceding the 14th month. From this point onwards, until month 18, total FAA concentration remained stable, and the content decreased at the end of the processing (between months 22 and 24).

## 1. Introduction

Dry-cured ham from pig has been part of the Mediterranean diet since ancient times. The quality of dry-cured ham is determined by the interactions between the characteristics of the raw meat and the biochemical changes that occur during processing [1]. Besides water loss and salt diffusion, the characteristics of the final product are greatly dependent on the presence and evolution of enzymatic activity [2], mainly lipolysis and proteolysis phenomena and Maillard and Strecker reactions. In this regard, there is a hydrolysis of tri-acylglycerols and phospholipids by lipases, esterases, phospholipases and lysophospholipases [3], followed by oxidative reactions that produce aroma volatile compounds, conforming the flavour of the dry-cured ham [4]. On the other hand, proteolysis contributes to texture through the breakdown of the muscle structure, to taste through the generation of peptides and free amino acids, and to the aroma by further degradation of free amino acids [2]. Free amino acids (FAA) are generated during ham processing from muscle peptides and exogenous exopeptidases, and their increase may be influenced by several parameters such as residual proteolytic enzyme activities, product composition, temperature, pH, and substrate availability [5].

The novelty of this work consists of the use of meat from the Chato murciano (CM) (a native pig breed from Murcia, Spain) for manufacturing dry-cured ham, as products from this breed have not been the subject of many studies until now. CM meat exhibits different characteristics to commercial genotype meat [6]. In addition, derived products with clearly distinctive qualities are obtained from the native pig breeds of southern Europe [7]. For this reason, CM dry-cured products are well accepted by consumers [8]. Previous works have described the fatty acids and lipolysis of CM dry-cured loin [9,10]. In addition, the percentages of fatty acids from intramuscular fat in dry-cured ham have been previously reported [8], although the concentration values for the different lipid fractions have not. A greater knowledge of the fatty acid composition of each lipid fraction and the free amino acid (FAA) profiles of dry-cured ham is necessary, since this is essential in order to improve processing and ensure a genuine product from a native pig breed.

Consequently, the purpose of this work was to characterise the fatty acid composition of the neutral and polar lipid fractions, as well as free fatty acid concentration of the intramuscular fat, and the free amino acid changes throughout the manufacturing of CM dry-cured ham.

## 2. Materials and Methods

### 2.1. Raw Materials and Dry-Cured Ham Manufactured

Twenty-four pure CM pigs dry-cured hams were analysed. Thighs were removed from the carcass and manufactured in a factory following the traditional method as described Salazar et al. (2014) [8]. The mean weight of the fresh hams was 12.28 kg ± 1.10. Sampling was done in raw meat, and at 14, 18, 22 and 24 months of processing (ripening stage).

Samples were obtained from the central part of the piece by extracting a cylinder (sized 10 × 2.5 cm) with a stainless steel tube with a cutting edge, including the *Semimembranosus*, *Biceps femoris* and *Semitendinosus* muscles (‘maza’). After sampling at 14, 18, 22 and 24 months of manufacturing, hams were stuffed with lard, to allow for ripening. The collected samples were stored at −80 °C until analysis. Samples were analysed in triplicate.

### 2.2. Fatty Acids Determination

Intramuscular fat was obtained from dry-cured ham according to Folch et al. (1957) [11]. NH2-aminopropyl mini-columns were used to obtain neutral lipids (NL), polar lipids (PL) and free fatty acids (FFA) from the lipid extract, following the method described by Ruiz et al. (2004) [12]. Fatty acid methyl esters (FAME) of each fraction were prepared using the procedure described by Sandler and Karo (1992) [13].

Identification and quantification of FAME were based on retention times and peak area integration of the reference compounds (Sigma, St. Louis, MO, USA) obtained in a Shimadzu (GC 2010) gas chromatograph, equipped with a flame ionization detector (FID), an injector on column and a fused-silica capillary column (Supelco omega wax, Supelco, Bellefonte, PA, USA) (30 m length, 0.25 mm internal diameter and 0.25 µm film thickness), as reported in Salazar et al. (2016) [9] and Tejada et al. (2016) [10]. Solutions between 0.01 mg/mL and 25 mg/mL were prepared for: C14:0, C16:0, C16:1, C18:0, C18:1, C18:2, C18:3, C20:0 and C20:4. C19:0 was used as internal standard.

### 2.3. Free Amino Acids Determination

Free amino acid content was analysed according to Abellán et al. (2018) [14]. FAA extract was pre-column derivatisated with ortho-phthaldialdehyde (OPA) according to Jones et al. (1981) [15]. Norvaline was added as internal standard before derivatisation. The OPA derivates were analysed in a HPLC (Shimadzu LC-10AD), equipped with a fluorescence detector (RF-10A XL) and an eclipse XDB-C18 column (5 µm / 4.6 mm × 250 mm).

Individual FAA were identified and quantified by comparing their retention times with those of authenticated standards (Sigma, St. Louis, MO, USA). L-amino acids standard solutions between 1.75 mg/L and 28 mg/L were prepared of the following: aspartic acid (Asp), glutamic acid (Glu), asparagine (Asn), serine (Ser), threonine (Thr), glycine (Gly), arginine (Arg), alanine (Ala), tyrosine (Tyr), histidine (His), methionine (Met), tryptophan (Trp), valine (Val), phenylalanine (Phe), isoleucine (Ile), leucine (Leu) and lysine (Lys).

### 2.4. Statystical Analysis

The statistical evaluation of the experimental data was performed using the SPSS version 21.0 (IBM Corporation, Armonk, NY, USA) [16]. One-way ANOVA procedure and Tukey’s test were performed to determine the effect of time on fatty acids and free amino acids evolution in dry-cured ham throughout processing.

## 3. Results and Discussion

### 3.1. Total Fatty Acid Composition

Table 1 shows the total content of fatty acids in each lipid fractions of CM dry-cured ham at different processing times.

The concentrations of fatty acids in NL, PL and FFA are within the levels reported in Duroc [17] breed. Lower values of FFA were observed in dry-cured ham from Duroc and Large White breeds [18], probably due to the fact that dry-cured hams have been studied under different processing times being shorter the total ripening time in the previously cited study.

Processing time affects fatty acid content in all lipid fractions, showing a NL (*p* < 0.001) and PL (*p* < 0.001) decrease, and a FFA increase (*p* < 0.001). A similar evolution was observed in dry-cured ham from other breeds [17,19], as well as in CM dry-cured loin [8]. The main changes take place until month 18 of processing, showing variations of −40%, −88% and +516% in NL, PL and FFA respectively. After 22 months, the hydrolysis of NL, PL is not very intense, decreasing the release of free fatty acids, probably due to the decrease in lipase activity. In fact, the highest level of neutral and acid lipase activity takes place at 6 months of processing [3], and the highest level of phospholipase activity at post-salting stage [20] in Iberian dry-cured ham.

It has been reported that NL hydrolysis provides the main FFA in other native breeds such as the Iberian [21] and the Corsican [22], due to the fact that NL are the most abundant components in quantitative terms [23]. Nevertheless, NL lipolysis is lower in Bayona, Parma and Serrano dry-cured ham, where PL have been reported as the main subtracts of the lipid hydrolysis [3,24].

As a consequence of the lipolysis phenomena, NL decreases from 95.43% to 83.38% and PL from 2.57% to 0.41%. On the other hand, FFA increases from 2% to 16.21%. Duroc [17] and Iberian dry-cured ham [25] show percentages of lipid fractions of 87%, 0.8% and 11.3% and 92%, 4.5% and 3.5%, respectively, at the end of the processing. Lipolysis has a great effect on the organoleptic characteristics of CM dry-cured ham. In fact, a positive correlation has been established between odour intensity, flavour intensity, after-taste, cured flavour and total FFA content (0.61; 0.67; 0.71, 0.64; *p* < 0.05), showing the high influence of lipolysis on these sensory attributes (reported in Salazar et al. 2014 [8]).

### 3.2. Fatty Acid Composition of Neutral Lipids Fraction

Table 2 shows the concentrations of fatty acids in the neutral lipid (NL) fraction of CM dry-cured ham throughout processing.

The concentration of oleic acid (C18:1) is the highest throughout processing, according to results obtained in Duroc [17], constituting 47% of the total NL fatty acid content. Palmitic (C16:0), stearic (C18:0) and linoleic (C18:2) acids are the next in quantitative terms (22%, 11% and 11% of NL, respectively).

The main fatty acids in CM dry-cured ham are similar to those described in Iberian [19]. The fatty acids with the lowest concentration are palmitoleic acid (C16:1), arachidonic acid (C20:4), myristic acid (C14:0), linolenic acid (C18:3) and arachidic acid (C20:0) which, together, represent a total percentage close to 8% in NL.

The concentrations of all fatty acids except C20:4 decrease significantly (with percentages of decrease between 29% and 41%) in the first 18 months of ripening. The largest decreases observed during the first 18 months suggest a high lipolytic activity. The high lipolysis that takes place in NL during the processing of dry-cured ham has also been observed in CM dry-cured loin [9] and in Iberian dry-cured ham [19].

The concentrations of saturated fatty acids (SFA), monounsaturated fatty acids (MUFA) and polyunsaturated fatty acids (PUFA) of NL decrease during processing (*p* < 0.01; *p* < 0.001; *p* < 0.05, respectively), suggesting the occurrence of important lipolytic changes. That decrease gives rise to a parallel increase in FFA amount during processing [19].

A MUFA > SFA > PUFA relation is observed throughout processing, in accordance with previous studies published on Iberian dry-cured ham [19].

The percentages of MUFA, SFA and PUFA in CM dry-cured ham are 51%, 35% and 14%, respectively, at the end of the processing, while in Iberian dry-cured ham, these are 57%, 35% and 7% [21]. Similar values were reported in raw meat from the Celta pig [26].

CM dry-cured ham is an important source of PUFA and MUFA, which have been related to a lower risk of cardiovascular diseases [27], one of the main benefits of the Mediterranean diet.

### 3.3. Fatty Acid Composition of Polar Lipids Fraction

Table 3 shows the concentrations of fatty acids in the polar lipid (PL) fraction of CM dry-cured ham throughout processing.

A high variability is observed, with the main fatty acid changing each month throughout processing. Thus, C18:2 is the most abundant fatty acid in raw meat, followed by C16:0 and C18:1, as reported in Celta pig [26]; whereas C18:0, followed by C16:0 and C18:1, show higher values in dry-cured ham at month 24 of processing.

All fatty acids in the PL fraction except C16:1 and C20:0 (*p* > 0.05) decrease significantly during the manufacture process. The decrease is over 80% in all cases, with the exception of C18:0, which is 70%. It should be noted that C18:2 and C18:1 decrease by 94% and 86%, respectively. The fatty acids C18:3 and C20:0 are not detected at month 14 of processing. The results obtained in CM are similar to those described in Iberian [19] and French dry-cured ham [28]. The main PL degradation is observed during the first few months of processing, probably due to the higher phospholipase activity that takes place during those first stages [20,28]. Phospholipids are the most degraded lipid fraction in dry-cured ham obtained from the Duroc manufacturing process [17], in contrast to the present study, where the most degraded are NL.

The PUFA > SFA > MUFA relation (47%; 34.54% and 18.46%, respectively) is observed at the beginning of the manufacture process (day 0). Similar percentages have been reported in Celta dry-cured ham [26], although PUFA are slightly lower (41.53% PUFA, 38.39% SFA and 22.23% MUFA). The relation is SFA > PUFA > MUFA (54.14%; 24.63% and 21.22%, respectively) at month 24 of processing. PUFA decrease by 93.55% (*p* < 0.001), SFA by 80.85% (*p* < 0.001) and MUFA by 86.31% (*p* < 0.001). In all cases, the decrease is significant (*p* < 0.05) between day 0 and month 18 of processing. It has been previously reported that PL have a higher oxidation tendency than NL [29] due to the high PUFA content. These fatty acids undergo higher oxidation due to their chemical structure and their location in the membranes, next to pro-oxidant substances such as heme pigment [30].

### 3.4. Free Fatty Acids

Table 4 shows the concentrations of free fatty acids (FFA) in CM dry-cured ham throughout processing.

As in the NL fraction, the main fatty acids are C18:1, C16:0, C18:2 and C18:0 (these represent 45%, 20%, 16% and 11%, respectively). The C18:2, C16:0 and C18:0 are the main FFA (in that order), reported in dry-cured ham manufactured from Duroc and Large White pigs, but the concentrations are lower than in CM [18]. The C18:1, C18:2, C16:0 and C18:0 are the main FFA in raw meat from the Celta pig [26]. Those FFA with lower concentrations in CM dry-cured ham are C16:1, C20:4, C14:0, C18:3 and C20:0, which represent around 8% of the total FFA content, a similar percentage to that observed in NL.

The concentration of all FFA studied increase significantly during processing, with the exception of C20:0 (*p* > 0.05). These results are similar to those previously reported by Flores et al. (2009) [20] for Iberian dry-cured ham, with a total processing time of two years and with Serrano dry-cured ham [18]. As a general rule, the greatest FFA increase takes place throughout the first 18 months of processing. This fact suggest a high lipolytic activity during the first stages and an oxidation of fatty acids released from NL and PL from this moment, since the FFA fraction of dry-cured hams depends both on the release of fatty acids from the NL and PL and the oxidation of these fatty acids [17].

Significant changes are not observed between month 18 and the end of processing (month 24). It is probable that a decrease of the lipolytic enzyme activity on progressive dehydration and salt content at the end stages could also occur [21]. A significant increase of C20:4 is observed (*p* < 0.01) up to month 22 as a result of the hydrolysis of phospholipids, since this fatty acid is not modified during the processing in the NL fraction.

Regarding SFA, MUFA and PUFA concentrations, these increase significantly throughout processing (*p* < 0.01; *p* < 0.001; *p* < 0.05). The MUFA>SFA>PUFA relation is observed during the whole manufacturing process (36.26%, 48.02% and 15.72%, at day 0; and 32.24%, 48.16% and 19.59%, respectively, at 24 months), as reported in Iberian dry-cured ham [19,20]. A positive correlation was established between rancidity (reported in Salazar et al. 2014 [8]) and PUFA and MUFA (0.71 and 0.61, respectively; *p* < 0.05), as a consequence of the higher oxidation of these fatty acids.

The results observed in lipid fractions of CM dry-cured ham imply that, besides the NL, the PL also provide FFA to the final product although, as discussed above, the main contribution comes from the first fraction, because they are quantitatively the most abundant in intramuscular fat.

### 3.5. Free Amino Acids

Table 5 shows the evolution of free amino acids (FAA) in CM dry-cured ham throughout processing.

The total FAA concentration in CM dry-cured ham is similar to that reported in Celta dry-cured ham (8000 mg/100 g dry matter) [31]. However, higher concentration values have been observed for other types of dry-cured hams (around 12500 mg/100 g dry matter) by Pérez-Palacios et al. (2010) [32]. On the other hand, lower FAA concentrations (around 4000 mg/100 g dry matter) have been reported by del Olmo et al. (2013) [33]. The differences in FAA levels of dry-cured ham are due to the diverse processing conditions that have an influence on enzymatic activity, as well as the different proteolytic activity of each pig breed, and the ages at which the pigs are slaughtered [34].

The most abundant FAA in the raw pieces (at day 0) is Arg, followed by His, Ala and the combinations of Thr + Gly. The amino acid Ala is also among the main FAA in Iberian [32] and Celta dry-cured ham [31].

At the end of processing, the FAA that shows that the highest concentration is Glu followed by Arg, Leu and Lys, which basically coincides with those observed in different types of dry-cured ham [31,33]. Cys is not detected, which is in line with previous studies for Iberian [32] and Serrano dry-cured ham [33].

Glu (the main amino acid in CM dry-cured ham) is the amino acid with the greatest impact on meat flavour [35] and it has been linked with the ‘umami’ taste. Thus, Glu will contribute significantly to the taste properties of CM dry-cured ham, since its concentration is above the threshold of sensory perception (300 mg/L) [36]. Other dry-cured ham show lower concentrations of Glu than the CM [33].

Regarding processing time, the concentration of all FAA detected increases significantly (*p* < 0.001) up to month 14, remaining constant from this moment until month 18 of the total processing time (*p* > 0.05). A significant increase until month 15 of processing has also been reported in Serrano dry-cured ham [33].

Asn, Ser, Arg, Tyr, His and Lys show a significant decrease during the last months of processing. The concentration values of Asp, Glu, Thr + Gly, Ala, Met, Trp, Val, Phe, Ile and Leu, also decrease throughout this stage, although the decrease is not statistically significant (*p* > 0.05). Some authors also found a decrease in FAA during the extended maturing of Iberian dry-cured ham [37]. However, no FAA decrease in the last stages of processing has been observed in other dry-cured hams with shorter maturing periods [31,32]. There is no evidence of a decrease in FAA concentrations in Italian dry-cured ham [38], even though it has a very similar total processing time to CM.

FAA liberated by protein hydrolysis undergo a Maillard reaction and Strecker degradation at the end of the dry-cured ham manufacturing process [37]. This fact could explain the decrease in FAA levels in CM dry-cured ham. Therefore, the evolution of FAA concentration in CM dry-cured ham throughout processing depends on the relationship between formation and degradation, meaning that the decrease in FAA levels at the end of the processing reflects the fact that degradation of these compounds is more active than their production during this period. This has been attributed to several phenomena. Protease activity is higher at the beginning of the process; for example, alanyl aminopeptidase is the enzyme responsible of the hydrolysis of a wide range of amino acids for the amino terminus of the protein, and it is active for the first 240 days of processing [37]. The exopeptidase activity is lower at the end of processing due to the inhibitory effect of salt, dehydration, and own enzyme proteolysis. In addition, FAA themselves could be inhibitors of proteolytic phenomena [39]. Furthermore, some of the amino acids released undergo oxidative degradation during the drying and maturing stages, leading to the formation of volatile compounds [24]. This fact indicates that FAA concentration decreases during last months of processing. Those volatile compounds are responsible of the dry-cured ham’s flavour. In fact, a positive correlation has been established between total FAA content and flavour intensity (0.61; *p* < 0.05), and between most of the individual FAA, mainly with Lys, Arg and His (0.71; 0.71, 0.64; *p* < 0.05) [8].

## 4. Conclusions

It has been found that processing time has a great effect on fatty acids and free amino acids in CM dry-cured ham. The evolution and the concentration are similar to those of other native pig breeds.

CM dry-cured ham lipolysis occurs mainly during the first 18 months of processing, showing a decrease in NL and PL fractions, and an increase in the fraction of FFA. MUFA are the majority in the NL and FFA fractions throughout all the periods studied. In the PL fraction, the fatty acid profile changes during processing. PUFA are the majority at first and SFA at the end of processing. MUFA are the minority in the phospholipid fraction throughout the whole processing time.

On the other hand, the release of amino acids occurs between month 14 and month 18, with a decrease in most amino acids studied from this point until month 24. Therefore, CM dry-cured ham is a product that supports long maturation times, unaffected by intense proteolysis. Glu is the main amino acid, followed quantitatively by Lys, Ala, Leu and Arg.

The biochemical CM dry-cured ham changes contribute to the typical flavor development of this product, high valued by consumers in the Mediterranean area.

## Figures and Tables

**Table 1:** Total fatty acids contents of the intramuscular lipid fractions of Chato murciano dry-cured ham. Results are expressed as g fatty acid methyl esters/Kg of intramuscular fat (means values ± SEM).

Lipid Fraction	Processing Time					*p*-Value
	Day 0 (*n* = 24)	14 Months (*n* = 24)	18 Months (*n* = 24)	22 Months (*n* = 24)	24 Months (*n* = 24)	
Neutral lipids (% total)	871.1 ± 57.2 ^c^ (95.4)	750.1 ± 50.3 ^bc^ (91.4)	587.1 ± 24.3 ^ab^ (84.8)	493.8 ±35.3 ^a^ (82.1)	578.7 ± 42.6 ^ab^ (83.4)	<0.0001
Polar lipids (% total)	23.4 ± 0.6 ^c^ (2.57)	9.0 ± 0.2 ^b^ (1.1)	3.7 ± 0.4 ^a^ (0.54)	3.07 ± 0.4 ^a^ (0.5)	2.9 ± 0.2 ^a^ (0.4)	<0.0001
Free fatty acids (% total)	18.3 ± 0.7 ^a^ (2.0)	61.9 ± 6.3 ^b^ (7.5)	101.7 ± 5.4 ^c^ (14.7)	104.9 ± 2.2 ^c^ (17.4)	112.6 ± 7.0 ^c^ (16.2)	<0.0001

^a,b,c^ Values within a row with different superscripts differ significantly at *p* ≤ 0.05 (Tukey’s Test).

**Table 2:** Effect of processing time on fatty acids contents of neutral lipids in Chato murciano dry-cured ham. Results are expressed as g fatty acid methyl esters/Kg of intramuscular fat (means values ± SEM).

Fatty Acid	Processing Time					*p*-Value
	Day 0 (*n* = 24)	14 Months (*n* = 24)	18 Months (*n* = 24)	22 Months (*n* = 24)	24 Months (*n* = 24)	
C14:0	11.3 ± 0.4 ^bc^	12.4 ± 1.0 ^c^	7.0 ± 0.1 ^a^	5.7 ± 0.9 ^a^	7.2 ± 0.7 ^a^	<0.0001
C16:0	222.5 ± 4.8 ^c^	182.8 ± 11.7 ^bc^	136.2 ±0.5 ^ab^	111.0 ± 20.9 ^a^	131.9 ± 17.5 ^ab^	0.001
C16:1	32.1 ± 2.2 ^bc^	40.3 ± 3.3 ^c^	20.3 ± 2.2 ^a^	18.7 ± 3.6 ^a^	20.2 ± 3.0 ^a^	0.001
C18:0	107.1 ± 2.1 ^c^	69.5 ± 6.6 ^a^	68.4 ± 4.7 ^a^	52.1 ± 11.2 ^a^	65.0 ± 9.3 ^a^	0.004
C18:1	384.3 ± 41.2 ^c^	342.3 ± 36.5 ^b^	271.6 ± 17.4 ^a^	222.6 ± 27.9 ^a^	274.3 ± 29.9 ^a^	0.001
C18:2	91.7 ± 10.9 ^b^	81.9 ± 3.9 ^ab^	66.5 ± 4.5 ^a^	64.9 ± 15.0 ^a^	63.2 ± 9.3 ^a^	0.006
C18:3	4.5 ± 0.2 ^c^	3.7 ± 0.7 ^bc^	1.9 ± 0.1 ^a^	2.2 ± 0.4 ^ab^	2.1 ± 0.2 ^ab^	0.002
C20:0	1.4 ± 0.1 ^b^	0.9 ± 0.1 ^ab^	0.9 ± 0.0 ^a^	0.7 ± 0.1 ^a^	0.9 ± 0.1 ^a^	0.010
C20:4	16.3± 1.4 ^a^	16.1 ± 0.8 ^a^	14.2 ± 0.5 ^a^	15.7 ± 5.1 ^a^	13.9 ± 4.1 ^a^	0.96
ΣSFA	342.3 ± 7.1 ^c^	265.6 ± 16.8 ^ab^	212.6 ± 5.2 ^a^	169.5 ± 33.2 ^a^	205.0 ± 27.5 ^a^	0.001
ΣMUFA	416.4 ± 42.3 ^c^	382.6 ± 39.4 ^b^	291.9 ± 9.2 ^a^	241.3 ± 32.8 ^a^	294.5 ± 34.7 ^a^	0.008
ΣPUFA	112.4 ± 12.3 ^c^	101.8 ± 4.5 ^a^	82.6 ± 4.2 ^a^	82.9 ± 20.4 ^a^	79.2 ± 13.6 ^a^	0.041

SFA: saturated fatty acids; MUFA: monounsaturated fatty acids; PUFA: polyunsaturated fatty acids. ^a,b,c^ Values within a row with different superscripts differ significantly at *p* ≤ 0.05 (Tukey’s Test).

**Table 3:** Effect of processing time on fatty acids contents of polar lipids in Chato murciano dry-cured ham. Results are expressed as g fatty acid methyl esters/Kg of intramuscular fat (means values ± SEM).

Fatty Acid	Processing Time					*p*-Value
	Day 0 (*n* = 24)	14 Months (*n* = 24)	18 Months (*n* = 24)	22 Months (*n* = 24)	24 Months (*n* = 24)	
C14:0	0.2 ± 0.0 ^b^	nd	0.02 ± 0.03 ^a^	0.02 ± 0.02 ^a^	0.02 ± 0.02 ^a^	0.001
C16:0	5.3 ± 0.01 ^c^	2.1 ± 0.1 ^b^	1.0 ± 0.2 ^a^	0.1 ± 0.1 ^a^	0.7 ± 0.1 ^a^	<0.0001
C16:1	0.1 ± 0.0 ^a^	0.1 ± 0.1 ^a^	nd	nd	0.03 ± 0.03 ^a^	0.090
C18:0	2.7 ± 0.1 ^c^	1.6 ± 0.2 ^b^	0.7 ± 0.2 ^a^	0.8 ± 0.2 ^a^	0.9 ± 0.1 ^a^	<0.0001
C18:1	4.2 ± 0.3 ^c^	2.2 ± 0.1 ^b^	1.1 ± 0.1 ^a^	0.7 ± 0.0 ^a^	0.6 ± 0.1 ^a^	<0.0001
C18:2	8.1 ± 0.4 ^c^	2.5 ± 0.3 ^b^	0.6 ± 0.2 ^a^	0.6 ± 0.1 ^a^	0.5 ± 0.1 ^a^	<0.0001
C18:3	0.1 ± 0.0 ^b^	nd	nd	nd	nd	<0.0001
C20:0	0.02 ± 0.02 ^a^	nd	nd	nd	nd	0.452
C20:4	2.7 ± 0.2 ^b^	0.7 ± 0.2 ^a^	0.3 ± 0.1 ^a^	0.2 ± 0.1 ^a^	0.2 ± 0.1 ^a^	<0.0001
ΣSFA	8.1 ± 0.1 ^c^	3.7 ± 0.2 ^b^	1.8 ± 0.3 ^a^	1.5 ± 0.2 ^a^	1.6 ± 0.2 ^a^	<0.0001
ΣMUFA	4.3 ± 0.3 ^c^	2.3 ± 0.1 ^b^	1.1 ± 0.1 ^a^	0.7 ± 0.0 ^a^	0.6 ± 0.0 ^a^	<0.0001
ΣPUFA	11.0 ± 0.5 ^c^	3.1 ± 0.4 ^b^	0.9 ± 0.2 ^a^	0.8 ± 0.2 ^a^	0.7 ± 0.1 ^a^	<0.0001

nd: not detected. SFA: saturated fatty acids; MUFA: monounsaturated fatty acids; PUFA: polyunsaturated fatty acids. ^a,b,c^ Values within a row with different superscripts differ significantly at *p* ≤ 0.05 (Tukey’s Test).

**Table 4:** Effect of processing time on free fatty acids contents in Chato murciano dry-cured ham. Results are expressed as g fatty acid methyl esters/Kg of intramuscular fat (means values ± SEM).

Fatty Acid	Processing Time					*p*-Value
	Day 0 (*n* = 24)	14 Months (*n* = 24)	18 Months (*n* = 24)	22 Months (*n* = 24)	24 Months (*n* = 24)	
C14:0	0.3 ± 0.0 ^a^	0.7 ± 0.1 ^ab^	1.1 ± 0.2 ^bc^	1.2 ± 0.1 ^bc^	1.3 ± 0.1 ^c^	0.001
C16:0	3.9 ± 0.2 ^a^	12.5 ± 1.0 ^b^	17.1 ± 2.0 ^bc^	21.6 ± 0.7 ^cd^	23.6 ± 1.7 ^d^	<0.0001
C16:1	0.5 ± 0.0 ^a^	1.8 ± 0.0 ^a^	3.8 ± 0.9 ^b^	3.9 ± 0.3 ^b^	3.8 ± 0.1 ^b^	<0.0001
C18:0	2.4 ± 0.4 ^a^	7.3 ± 0.9 ^bc^	7.1 ± 0.8 ^b^	9.9 ± 0.8 ^bc^	11.3 ± 1.3 ^c^	<0.0001
C18:1	8.3 ± 0.4 ^a^	23.8 ± 2.3 ^ab^	39.6 ± 7.1 ^bc^	44.9 ± 4.1 ^c^	50.3 ± 2.2 ^c^	<0.0001
C18:2	2.3 ± 0.2 ^a^	11.6 ± 1.4 ^b^	14.3 ± 0.9 ^bc^	17.7 ± 1.7 ^c^	17.0 ± 1.2 ^bc^	<0.0001
C18:3	0.1 ± 0.0 ^a^	0.3 ± 0.0 ^ab^	0.5 ± 0.1 ^b^	0.5 ± 0.0 ^b^	0.5 ± 0.0 ^b^	<0.0001
C20:0	0.1 ± 0.0 ^a^	0.1 ± 0.0 ^a^	0.1 ± 0.0 ^a^	0.1 ± 0.0 ^a^	0.1 ± 0.0 ^a^	0.298
C20:4	0.4 ± 0.1 ^a^	3.8 ± 0.5 ^b^	3.6 ± 0.2 ^ab^	4.9 ± 1.1 ^b^	4.6 ± 1.1 ^b^	0.009
ΣSFA	6.6 ± 0.6 ^a^	20.6 ± 2.1 ^b^	29.5 ± 1.9 ^c^	32.9 ± 0.3 ^c^	36.4 ± 2.9 ^c^	<0.0001
ΣMUFA	8.8 ± 0.4 ^a^	25.5 ± 2.3 ^b^	51.3 ± 2.6 ^c^	48.8 ± 4.4 ^c^	54.1 ± 2.2 ^c^	<0.0001
ΣPUFA	2.8 ± 0.3 ^a^	15.7 ± 1.9 ^b^	20.7 ± 1.6 ^b^	23.3 ± 2.8 ^b^	22.2 ± 2.2 ^b^	<0.0001

SFA: saturated fatty acids; MUFA: monounsaturated fatty acids; PUFA: polyunsaturated fatty acids. ^a,b,c,d^ Values within a row with different superscripts differ significantly at *p* ≤ 0.05 (Tukey’s Test).

**Table 5:** Effect of processing time on free amino acids in Chato murciano dry-cured ham. Results are expressed as g /Kg of total solids (means values ± SEM).

Amino Acid	Processing Time					*p*-Value
	Day 0 (*n* = 24)	14 Months (*n* = 24)	18 Months (*n* = 24)	22 Months (*n* = 24)	24 Months (*n* = 24)	
Asp	0.2 ± 0.0 ^a^	4.1 ± 0.3 ^b^	3.7 ± 0.2 ^b^	3.5 ± 1.5 ^b^	3.4 ± 0.3 ^b^	<0.0001
Glu	0.4 ± 0.0 ^a^	13.2 ± 0.9 ^b^	11.4 ± 0.5 ^b^	10.4 ± 1.2 ^b^	10.2 ± 0.7 ^b^	<0.0001
Asn	0.1 ± 0.0 ^a^	0.4 ± 0.0 ^ab^	0.7 ± 0.1 ^b^	0.7 ± 0.1 ^b^	0.4 ± 0.1 ^ab^	<0.0001
Ser	0.3 ± 0.1 ^a^	0.9 ± 0.1 ^c^	0.9 ± 0.1 ^c^	0.4 ± 0.0 ^ab^	0.8 ± 0.1 ^bc^	<0.0001
Thr + Gly	0.7 ± 0.1 ^a^	2.3 ± 0.2 ^b^	2.4 ± 0.2 ^b^	2.4 ± 0.5 ^b^	2.4 ± 0.2 ^b^	<0.0001
Arg	2.9 ± 0.2 ^a^	7.4 ± 0.5 ^c^	7.4 ± 0.4 ^c^	5.2 ± 0.6 ^b^	5.5 ± 0.4 ^b^	<0.0001
Ala	1.0 ± 0.1 ^a^	7.9 ± 0.6 ^b^	7.5 ± 0.9 ^b^	8.2 ± 0.9 ^b^	9.1 ± 0.6 ^b^	<0.0001
Tyr	0.2 ± 0.0 ^a^	2.9 ± 0.2 ^bc^	3.2 ± 0.1 ^c^	2.5 ± 0.3 ^bc^	2.5 ± 0.1 ^b^	<0.0001
His	1.7 ± 0.2 ^a^	5.6 ± 0.3 ^b^	5.2 ± 0.4 ^b^	4.7 ± 0.6 ^b^	2.6 ± 0.2 ^a^	<0.0001
Met + Trip	0.6 ± 0.1 ^a^	2.9 ± 0.2 ^b^	3.6 ± 0.2 ^b^	3.6 ± 0.5 ^b^	3.5 ± 0.2 ^b^	<0.0001
Val	0.4 ± 0.1 ^a^	4.5 ± 0.3 ^b^	4.9 ± 0.4 ^b^	4.5 ± 0.7 ^b^	4.1 ± 0.2 ^b^	<0.0001
Phe	0.4 ± 0.1 ^a^	3.9 ± 0.2 ^b^	4.2 ± 0.2 ^b^	4.3 ± 0.6 ^b^	4.5 ± 0.5 ^b^	<0.0001
Ile	0.3 ± 0.0 ^a^	3.9 ± 0.3 ^b^	4.4 ± 0.2 ^b^	4.6 ± 0.7 ^b^	4.4 ± 0.2 ^b^	<0.0001
Leu	0.4 ± 0.1 ^a^	7.8 ± 0.5 ^b^	8.3 ± 0.4 ^b^	8.2 ± 1.0 ^b^	8.5 ± 0.5 ^b^	<0.0001
Lys	0.5 ± 0.1 ^a^	12.0 ± 0.8 ^bc^	13.1 ± 1.2 ^c^	8.8 ± 1.6 ^bc^	8.3 ± 1.0 ^b^	<0.0001
Total	10.4 ± 1.2 ^a^	81.2 ± 5.1 ^c^	81.0 ± 2.4 ^bc^	72.1 ± 9.6 ^b^	70.1 ±4.4 ^c^	<0.0001

^a,b,c^ Values within a row with different superscripts differ significantly at *p* ≤ 0.05 (Tukey’s Test).

## References

[1] Vestegaard C.S., Schivazappa C., Virgili R. (2000). Lipolysis in dry-cured ham maturation. Meat Sci..

[2] Toldrá F., Flores M. (1998). The role of muscle proteases and lipases in flavor development during the processing of dry-cured ham. Crit. Rev. Food Sci..

[3] Motilva M.J., Toldrá F., Nieto P., Flores J. (1993). Muscle lipolysis phenomena in the processing of dry-cured ham. Food Chem..

[4] Toldrá F., Aristoy M.C., Toldrá F. (2010). Dry-cured ham. Handbook of Meat Processing.

[5] Armero E., Basega M., Aristoy M.C., Toldrá F. (1999). Effects of sire type and sex on pork muscle exopeptidase activity, natural dipeptides and free amino acids. J. Sci. Food Agric..

[6] Poto A., Galián M., Peinado B. (2007). Chato murciano pig and its crosses with Iberian and large white pigs, reared outdoors. Comparative study of the carcass and meat characteristics. Livestig. Sci..

[7] Pugliese C., Sirtori F. (2012). Quality of meat and meat products produced from southern European pig breeds. Meat Sci..

[8] Salazar E., Abellán A., Cayuela J.M., Poto A., Girón F., Zafrilla P., Tejada L. (2014). Effect of processing time on the quality of dry-cured ham obtained from a native pig breed (*Chato murciano*). Anim. Prod. Sci..

[9] Salazar E., Abellán A., Cayuela J.M., Poto A., Tejada L. (2016). Dry-cured loin from the native pig breed Chato murciano with high unsaturated fatty acid content undergoes intense lipolysis of neutral and polar lipids during processing. Eur. J. Lipid Sci. Tech..

[10] Tejada L., Salazar E., Abellán A., Peinado B., Mulero J., Cayuela J.M. (2016). A comparison of fatty acid profiles and lipolysis during ripening of dry-cured loins obtained from a native pig breed (Chato murciano) and from a modern crossbreed pig. Anim. Prod. Sci..

[11] Folch J., Lees M., Stanley G. (1957). A simple method for the isolation of total lipides from animal tissues. J. Biol. Chem..

[12] Ruíz J., Antequera T., Andres A.I., Petron M.J., Muriel E. (2004). Improvement of a solid phase extraction method for anlysis of lipid fractions in muscle foods. Anal. Chim. Acta.

[13] Sandler S.R., Karo W. (1992). Source Book of Advances Organic Laboratory Preparations.

[14] Abellán A., Salazar E., Vázquez J., Cayuela J.M., Tejada L. (2018). Changes in proteolysis during the dry-cured processing of refrigerated and frozen loin. LWT Food Sci. Technol..

[15] Jones B.N., Pääbo S., Stein S. (1981). Amino-acid analysis and enzymatic secuence determination of peptides by an improved ortho-phthaldialdehyde pre-column labeling procedure. J. Liq. Chromatogr..

[16] (2012). IBM SPSS Statistics for Windows.

[17] Storrustløkken L., Devle H.M., Haseth T.T., Egelandsdal B., Naess-Andresen C.F., Hollung K., Berg P., Ekeberg D., Alvseike O. (2015). Lipid degradation and sensory characteristics of M. biceps femoris in dry-cured hams from Duroc using three different processing methods. Int. J. Food Sci. Tech..

[18] Del Olmo A., Calzada J., Nuñez M. (2013). Lipolysis, Lipid Peroxidation, and Color Characteristics of Serrano Hams from Duroc and Large White Pigs during Dry-Curing. J. Food Sci..

[19] Andrés A.I., Cava R., Martin D., Ventanas J., Ruíz J. (2005). Lipolysis in dry-cured ham: Influence of salt content and processing conditions. Food Chem..

[20] Flores M., Aristoy M.C., Antequera T., Barat J.M., Toldrá F. (2009). Effect of prefreezing ham on endogenous enzyme activity during the processing of Iberian dry-cured hams. Meat Sci..

[21] Martín L., Córdoba J.J., Ventanas J., Antequera T. (1999). Changes in intramuscular lipids during ripening of Iberian dry-cured ham. Meat Sci..

[22] Gandemer G. (2002). Lipids in muscle and adipose tissues, changes during processing and sensory properties of meat products. Meat Sci..

[23] Tejeda J.F., Gndemer G., Antequera T., Viau M., García C. (2002). Lipid traits of muscles as related to genotype and fattening diet in Iberian pigs: Total intramuscular lipids and triacylglycerols. Meat Sci..

[24] Buscailhon S., Berdague J.L., Monin G. (1993). Time-related changes in volatile compounds of lean tissue during processing of French dry-cured ham. J. Sci. Food Agric..

[25] Petrón M.J., Muriel E., Timón M.L., Martín L., Antequera T. (2004). Fatty acids and triacylglycerols profiles from different types of Iberian dry-cured ham. Meat Sci..

[26] Domínguez R., Martínez S., Gómez M., Carballo J., Franco I. (2015). Fatty acids, retinol and cholesterol composition in various fatty tissues of Celta pig breed: Effect of the use of chestnuts in the finishing diet. J. Food Compos. Anal..

[27] Hu F.B., Manson J.E., Willet W.C. (2001). Types of dietary fats and risk of coronary heart disease: A critical review. J. Am. Coll. Nutr..

[28] Buscailhon S., Monin G. (1994). Time-related changes in intramuscular lipids of lean tissue during processing of French dry-cured ham. Meat Sci..

[29] Igene J.O., Pearson A.M., Gray J.I. (1981). Effect of length of frozen storage cooking and holding temperatures upon components phospholipids and the fatty acid composition of meat triglycerides and phospholipids. Food Chem..

[30] Gray J.I., Pearson A.M., Pearson A.M., Dutson T.R. (1987). Rancidity and warmed-over flavour. Restructured meat and meat products. Advances in Meat Research.

[31] Bermúdez R., Franco D., Carballo J., Sentandreu M.Á., Lorenzo J.M. (2014). Influence of muscle type on the evolution of free amino acids and sarcoplasmic and myofibrillar proteins through the manufacturing process of Celta dry-cured ham. Food Res. Int..

[32] Pérez-Palacios T., Ruíz J., Barat J.M., Aristoy M.C., Antequera T. (2010). Influence of pre-cure freezing of Iberian ham on proteolytic changes throught the ripening process. Meat Sci..

[33] Del Olmo A., Calzada J., Gaya P., Nuñez M. (2013). Proteolysis, Texture, and Sensory Characteristics of Serrano Hams from Duroc and Large White Pigs during Dry-Curing. J. Food Sci..

[34] Rosell C.M., Toldrá F. (1998). Comparison of muscle proteolytic and lypolitic enzime levels in raw hams from Iberian and White pigs. J. Sci. Food Agric..

[35] Maga J.A., Shahidi F. (1998). Umami flavour of meat. Flavour of Meat Products and Seafood.

[36] Kato H., Rhue M.R., Nishimura T., Teranishi R., Buttery R.G., Shahidi F. (1989). Role of free amino acids and peptides in food taste. Flavor Chemistry, Trends and Developments.

[37] Toldrá F., Aristoy M.C., Flores M. (2000). Contribution of muscle aminopeptidases to flavor development in dry cured ham. Food Res. Int..

[38] Virgili R., Saccani G., Gabba L., Tanzi E., Bordini C.S. (2007). Changes of free amino acids and biogenic amines during extended ageing of Italian dry-cured ham. LWT Food Sci. Technol..

[39] Touraille C., Byle M.C. (1982). Influence du sexe et de l’age al’abattage sur les qualites organoleptiques des viandes de bovins Limousins abattus entre 16 et 33 mois. Bull. Tech. CRZ-V Theix INRA.

